# Effects of Sodium Hyaluronate Eye Drops With or Without Preservatives on Ocular Surface Bacterial Microbiota

**DOI:** 10.3389/fmed.2022.793565

**Published:** 2022-02-14

**Authors:** Yanlin Zhong, Xie Fang, Xuemei Wang, Yu-An Lin, Huping Wu, Cheng Li

**Affiliations:** ^1^Eye Institute & Affiliated Xiamen Eye Center, School of Medicine, Xiamen University, Xiamen, China; ^2^Fujian Provincial Key Laboratory of Ophthalmology and Visual Science & Ocular Surface and Corneal Diseases, Xiamen, China

**Keywords:** microbiome, ocular surface bacterial microbiota, preservatives, 16S rRNA gene amplicon sequencing, sodium hyaluronate eye drops, benzalkonium chloride

## Abstract

**Purpose:**

This study aimed to determine the composition and diversity of bacterial communities on the ocular surface before and after the intervention with sodium hyaluronate eye drops (with or without preservatives) using 16S rRNA gene amplicon sequencing.

**Methods:**

Sixteen healthy adults were randomly divided into two groups and treated with sodium hyaluronate eye drops with or without preservatives for 2 weeks. The individuals used the same artificial tears in both eyes. The microbial samples from the conjunctival sac of each participant were collected at baseline and 2 weeks after intervention. The diversity and taxonomic differences among different groups before and after intervention were compared by sequencing the V3–V4 region of the 16S rRNA gene.

**Results:**

The similarity in the binocular microbial community was high in 1 of the 16 volunteers (Bray-Curtis dissimilarity score < 0.3). At the genus level, 11 bacteria were detected in all samples with an average relative abundance of more than 1%. The bacterial community changed significantly after the use of sodium hyaluronate eye drops (with or without preservatives), whether within individuals or between individuals in different groups (*P* < 0.05, PERMANOVA). Different dosage forms of sodium hyaluronate eye drops significantly decreased the relative abundance of *Flavobacterium caeni* and *Deinococcus antarcticus*, respectively (*P* < 0.05).

**Conclusions:**

Healthy people had a rich diversity of the bacterial microbiota on the ocular surface, but the bacterial communities between the eyes were not completely similar. Irrespective of containing benzalkonium chloride (BAC), sodium hyaluronate eye drops can change the bacterial community on the ocular surface.

## Introduction

The ocular surface is a microecosystem in contact with the outside air and colonized by a specific microbial community (1). With the innovation of molecular biology techniques, such as polymerase chain reaction (PCR) and gene sequencing, it has been found that the total number of microorganisms is relatively small on the ocular surface compared with other parts of the body such as the intestinal tract, oral cavity, and skin, but they are quite abundant from the perspective of biodiversity (2–6). The ocular surface microbiota is mainly composed of bacteria, accounting for 98.15%, while fungi and viruses account for 0.94 and 0.91%, respectively (7). The ocular surface microbiota settles on the mucosal epithelium of the ocular surface and the tear film covering it. They form a dynamic balance microecosystem with the ocular surface tissue. The system is stable over time with low variability within an individual (8). However, differences exist among different individuals due to the influence of many factors. In healthy populations, age and region are the influencing factors for differences in microbial community (7, 9–12). Several studies have assessed the role of these microorganisms in ocular surface health and disease by characterizing ocular surface microbiota in patients under different disease conditions (13–19). Anthropogenic factors, such as the use of antibiotics (20) and the wearing of corneal contact lens (21, 22), also contribute to the changes in the microbiota on the ocular surface.

In recent decades, sodium hyaluronate eye drops have become an option for artificial tear therapy, thanks to sodium hyaluronate, a water-soluble polymer (23). Sodium hyaluronate not only has a strong water-retention function due to a large amount of negative charge, but also has high viscosity and affinity. It can stay on the ocular surface for a long time and lubricate the eye surface. It can also promote corneal epithelial extension and wound healing, reducing the adverse effects of preservatives in ophthalmic preparations (1). Sodium hyaluronate eye drops can be divided into single-dose and multi-dose types based on the presence of preservatives in them. Considering the risk of pollution, preservatives are added to many artificial tears to kill microbial vegetative forms and inhibit the growth of microorganisms for long-term preservation. Benzalkonium chloride (BAC) is the most common preservative. Several previous studies have found that BAC has a particular toxic effect on the ocular surface epithelium, and its toxicity is related to the frequency, concentration, tear secretion level, and severity of ocular surface diseases (24–27).

The ocular surface bacterial microbiota is the largest group in the ocular surface microbiota. Its colonization site is in the ocular surface epithelium and tear film, and therefore whether it is affected by the use of sodium hyaluronate eye drops is of great concern. Also, whether BAC affects the ocular surface bacterial community is not clear. To better understand the ocular surface bacteria and thus guide clinical medication, the present study aimed to (1) evaluate the diversity of ocular surface bacterial microbiota before and after the intervention with sodium hyaluronate eye drops using 16S rRNA gene amplicon sequencing and (2) compare the effects of different dose types with and without BAC on the diversity and composition of ocular surface bacterial microbiota.

## Materials and Methods

### Study Population and Trial Groups

This study was designed as a randomized, parallel-group, open-label, controlled trial. It included 16 healthy volunteers (10 women and 6 men, aged 28.25 ± 3.51 years). The participants all lived in the same city and were the medical staff of the Xiamen Eye Center. They had no symptoms of ophthalmic surface disease and no more than 12 points on the Ophthalmic Surface Disease Index (OSDI) screening questionnaire. They underwent detailed eye examinations on the day before sampling, including intraocular pressure, tear meniscus height, non-invasive breakup time, and lipid layer analysis. Table S1 shows the information of the included population and the measurements of ocular surface indicators. They did not have any systemic disease, ocular surface disease, uveitis, glaucoma, retinal disease, or a history of eye trauma/transplant; did not wear contact lenses; and did not use eye drops (artificial tears, antibiotics, steroids, and non-steroidal anti-inflammatory drugs) for 6 months. All the participants were randomly divided into two groups, each consisting of five women and three men. Table 1 shows the statistical data of the included population; no statistically significant difference was found in the indicators between the two groups (*P* > 0.05, Mann–Whitney *U* test). The first group was given 0.3% sodium hyaluronate eye drops containing preservatives (Santen, Osaka, Japan), four times a day for 2 weeks in both eyes. The second group was given preservative-free 0.3% sodium hyaluronate eye drops (Santen, Osaka, Japan) with the same frequency and duration as the first group. The study plan was approved by the ethics committee of Xiamen Eye Center affiliated to the Xiamen University (Ethics Number: xmykzx-ky-2020-010). The study followed the principles of the Declaration of Helsinki. All participants provided a written informed consent document, and the participation was voluntary.

**Table 1:** Demographic and clinical characteristics of the cohort.

	**All participants**	**Preservative-free group**	**Preservative-containing group**	*P*-value^*^
No. of participants, *n*	16	8	8	–
Age, median (range)	29 (22-33)	29.5 (25-33)	27 (22-33)	ns
Female, % (*n*)	62.5 (10)	62.5 (5)	62.5 (5)	ns
Eyes, *n*	32	16	16	–
Ethnicity	Chinese (100%)	Chinese (100%)	Chinese (100%)	ns
IOP, mean ± SD	14.27 ± 2.73	13.89 ± 2.37	14.65 ± 3.08	ns
TMH, mean ± SD	0.23 ± 0.03	0.24 ± 0.03	0.23 ± 0.03	ns
NIBUT, mean ± SD	13.94 ± 2.55	14.04 ± 2.59	13.84 ± 2.59	ns
Avg ICU, mean ± SD	77.47 ± 11.37	75.75 ± 11.02	79.19 ± 11.80	ns
OSDI, mean ± SD	3.44 ± 2.00	3.25 ± 1.98	3.63 ± 2.13	ns

*ICU, interferometric color units; IOP, intraocular pressure; NIBUT, non-invasive breakup time; ns, not significant; OSDI, ocular surface disease index; TMH, tear meniscus height*.

*Data presented as % (n) or mean (SD) or median (range)*.

* *P-values based on Mann–Whitney U test comparing the preservative-free and preservative-containing groups*.

### Sample Collection

The researchers took samples in a clean eye treatment room disinfected with ultraviolet light. The first sampling was done in the morning after the eye examination. The eyes were then treated with artificial tears and sampled again at the same time 2 weeks later. Based on the study by Shin et al. (22), topical anesthetics themselves could affect sequencing results. However, considering the previous reports of the differentiation between the superficial and deep microbiome (6), we used a single dose of anesthetic before sampling. Topical anesthetics allowed the subjects to cooperate fully by reducing blink reflexes and the discomfort of sampling. This ensured that sufficient and consistent pressure was taken during the sampling process to obtain high-quality samples. Meanwhile, it avoided the sample contamination caused by the accidental contact of the swab with the eyelid margin or the cornea, as well as the corneal epithelial injury caused by subjects' overreaction to the discomfort of sampling. After topical anesthesia with Proparacaine Hydrochloride Eye Drops (Alcon, TX, USA), a disposable sterile flocking cotton swab was used to wipe the upper and lower conjunctival sacs from the nasal side to the temporal side in a clockwise direction, and the intensity of the wiping was determined to avoid obvious discomfort. Care was taken not to touch the cornea, eyelid margin, or even eyelid skin throughout the process. Immediately after the collection, the swab was placed in a 1.5-mL sterile centrifuge tube (Axygen, CA, USA) and stored in an ultra-low temperature refrigerator at −80°C prior to DNA extraction, PCR amplification, and 16S rRNA gene amplicon sequencing (Guangzhou Genedenovo Biotechnology Co., Guangzhou, China). To avoid contamination, topical anesthetics were injected into disposable sterile swabs and placed in sterile centrifuge tubes as blank controls.

### DNA Extraction, PCR Amplification, and 16S rRNA Gene Amplicon Sequencing

The sample DNA was extracted using a HiPure soil DNA kit (Magen, Guangzhou, China) following the manufacturer's protocols. The blank samples were also fully extracted to exclude false-positive results from the process.

The V3–V4 region of 16S ribosomal RNA gene was amplified by PCR (94°C for 2 min, followed by 30 cycles at 98°C for 10 s, 62°C for 30 s, and 68°C for 30 s, and finally extended at 68°C for 5 min). Primers 341 F (5′- CCTAGGGNGGCWGCAG-3′) and 806 R (5′-GGACTACHVGGGTATCTAAT-3′) were used. PCR reactions were performed in triplicate in a 50-μL mixture containing 5 μL of 10 × KOD buffer, 5 μL of 2 mM dNTPs, 3 μL of 25 mM MgSO_4_, 1.5 μL of each primer (10 μM), 1 μL of KOD polymerase, and 100 ng of template DNA. The related PCR reagents were from Toyobo, Japan.

Amplicons were extracted from 2% agarose gels and purified using an AxyPrep DNA Gel Extraction Kit (Axygen Biosciences, CA, USA) following the manufacturer's protocols and quantified using an ABI StepOnePlus Real-Time PCR System (Life Technologies, CA, USA). Purified amplicons were pooled in equimolar amounts and paired-end sequenced (PE250) on an Illumina platform following the standard protocols. The raw sequence data were deposited into the Sequence Read Archive database (Accession Number: PRJNA720296).

### Processing of 16S rDNA Dataset

Raw reads were further filtered using FASTP (28) (version 0.18.0) to obtain high-quality clean reads, according to the following rules: (1) removing reads containing more than 10% of unknown nucleotides (N) and (2) removing reads containing <50% of bases with quality (Q-value) <20. Paired-end clean reads were merged as raw tags using FLASH (version 1.2.11) with a minimum overlap of 10 bp and mismatch error rates of 2% (29). Noisy sequences of raw tags were filtered using the QIIME (30) (version 1.9.1) pipeline under specific filtering conditions (31) to obtain high-quality clean tags. The filtering conditions were as follows: (1) raw tags were broken from the first low-quality base site where the number of bases in the continuous low-quality value (default quality threshold, ≤3) reached the set length (default length, 3); (2) subsequently, tags whose continuous high-quality base length was <75% of the tag length were filtered. Clean tags were searched against the reference database (version r20110519, http://drive5.com/uchime/uchime_download.html) to perform reference-based chimera detection using the UCHIME algorithm (32). All chimeric tags were removed, and the finally obtained effective tags were used for further analysis. The UPARSE (33) (version 9.2.64) pipeline was used to cluster all effective tags, and the sequences with 97% similarity were grouped into operational taxonomic units (OTUs) (33). In each OTU, the absolute abundance of tags was calculated. The tag sequence with the highest abundance was selected as a representative sequence within each cluster. The representative sequences were classified into organisms by a naive Bayesian model using an RDP classifier (34) (version 2.2) based on the SILVA (35) database (version 132), with the confidence threshold value of 0.8.

### Diversity and Statistical Analysis

The alpha diversity and beta diversity data of all populations before and after treatment were obtained through calculation and analysis. Observed-species index, Shannon index, and all other alpha diversity indices were calculated in QIIME (version 1.9.1) (Table S2). Rarefaction curves of alpha index and the stacked bar plot of the community composition were visualized in R project ggplot2 package (version 2.2.1). Unweighted UniFrac distance matrices were generated using the GUniFrac package (36) (version 1.0) in the R project. Bray-Curtis distance matrix was calculated in the R project Vegan package (version 2.5.3). Based on the distance index between samples, the Vegan package in the R project was used for the unweighted pair group method with arithmetic mean analysis (UPGMA), and the samples with smaller distance were merged into the same cluster. According to the study of Zilliox et al. (15), the similarity in ocular surface bacterial microbiota was high for Bray-Curtis dissimilarity scores <0.30. The principal coordinate analysis (PCoA) of distance index and the statistical analysis of Adonis (also known as PERMANOVA) test were performed in the R project Vegan package (version 2.5.3) and plotted in the R project ggplot2 package (version 2.2.1). We used PCoA to evaluate the ocular surface microbiome at baseline and the differences in repeated samples after intervention. In GraphPad Prism (version 9.2.0), the box diagram was used to show the distribution of the relative abundance of bacteria in different samples. The top 10 at the phylum level and the top 20 at the genus level were shown. Kruskal–Wallis test and one-way analysis of variance were used to compare the differences in alpha diversity index and distance index before and after intervention in different groups. A scatter plot was drawn to visualize the average relative abundance of “core” bacteria at the genus level in each group after intervention. Based on the abundance and frequency of species in the sample, the indicator analysis was used to calculate the indicator value (IndVal) of each species in each group. The higher the value of IndVal, the more likely the species was to be the indicator species of the group. The labdsv package in the R project was used to calculate the indicator value of the species whose abundance value was >0 and the total proportion was >0.1% in each sample of the comparison group. We used cross-validation test to get the *P*-value. The biomarker of each group, shown in the bubble chart, could be found intuitively through the bubble size.

## Results

### Sample Quality

**Figure 1:**
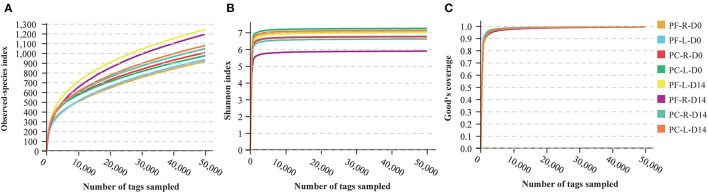
Rarefaction curves computed for alpha diversity indices. **(A)** Observed-species index for reflection of the observed OTUs of individual sample. **(B)** Shannon indices for the species richness of samples. **(C)** Good's coverage reflected the sequencing saturation of samples. All rarefaction curves of samples from the volunteers reached the saturation platform, indicating that the sequencing data size was reasonable.

The blank control samples used for monitoring DNA contamination did not produce 16S rRNA gene amplification products, indicating that the sampling process was controlled effectively. Then, 16S rDNA data were generated from 64 samples from 16 participants. A total of 6,870,617 reads were generated, with an average of 107,353.4 reads per sample. A total of 5,825,884 effective tags were generated after strict quality control of removing low-quality reads, tag mosaic, tag filtering, and tag de-chimerism. The total number of OTUs at 97% sequence similarity ranged from 674 to 2,127, with an average of 1,205.22 OTUs per sample and a total of 77,134 OTUs in all samples (Table S3). Rarefaction curves can directly reflect the rationality of sequencing data size by showing the variation trends in alpha diversity indices through a simulated resampling process and estimating the diversity of species in the environment. The curves reaching the saturation platform indicate that the sequencing data size is reasonable. The speed of the curve reaching the platform and the height of the curve reflect the difference in community alpha diversity among different groups. The value of Good's coverage equal to 1 indicates that all species in the sample have been detected. With the increase in the sequencing depth, the values of the Observed-species index, Shannon index, and Good's coverage increased, and later the curves gradually flattened (Figure 1). When the sequencing depth reached 50,000 tags/sample, these curves reached the saturated platform, and the average Good's coverage of all samples was 0.994 (Table S2), indicating that the sequencing had covered almost all species. The number of effective tags in this study was 91,029.44 tags/sample (Table S3), which showed that the depth of sequencing was sufficient for further analysis.

### Composition Analysis of Ocular Surface Bacterial Microbiota

**Figure 2:**
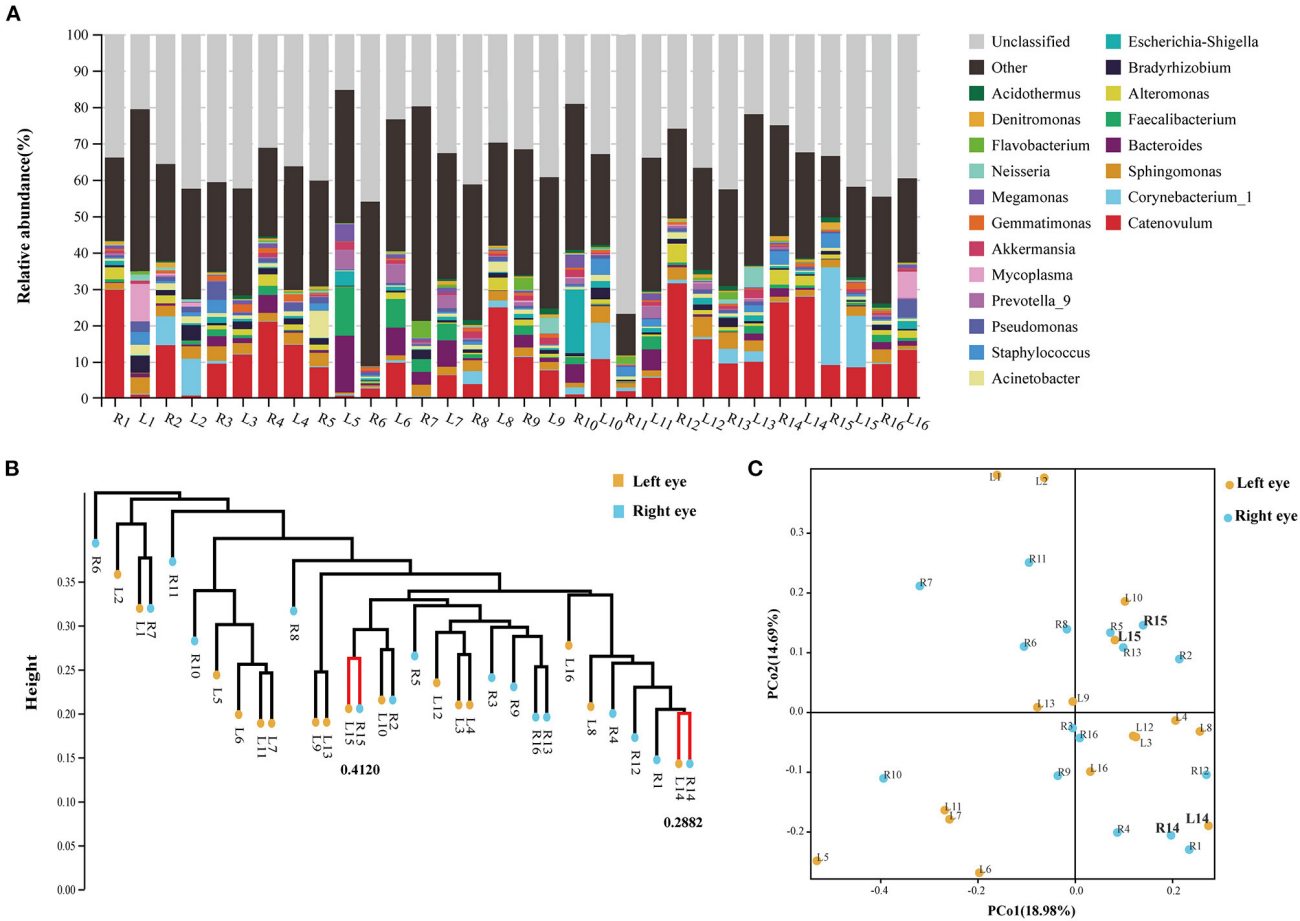
Analysis of composition and dissimilarity of bacterial community between eyes within individual at baseline. **(A)** Stacking map of abundance of bacteria at baseline. 16S rRNA gene sequences were classified into genus levels. **(B)** Unweighted pair group method with arithmetic mean analysis (UPGMA) of all samples at baseline. **(C)** PCoA analysis between right (R) and left (L) eyes.

The results of each sample were first plotted to see whether a single individual had similar bacterial microbiota in both eyes before the artificial tears were used (Figure 2A). In the UPGMA analysis (Figure 2B), the samples with smaller distances between the eyes of volunteers 14 and 15 were merged into the same cluster. According to the study by Zilliox et al. (15), the Bray-Curtis dissimilarity score < 0.3 was considered to be high in similarity between the two samples. In our study, only 1 of the 16 volunteers had a high similarity in the microbiome between eyes (Figure 2B, Volunteer 14, Bray-Curtis dissimilarity score <0.3). Using the single-sample *t*-test, the binocular Bray-Curtis dissimilarity score was compared with the hypothetical value (the hypothetical value was set to 0.3). The average value of the sample was 0.6133, which was significantly different from the hypothetical value (Figure S1, *P* < 0.0001). The PCoA plot showed that none of the paired samples between eyes had an identical microbial composition (Figure 2C). Therefore, our study showed that the composition of ocular surface microbial community was not completely consistent between eyes and between different individuals. One volunteer had mainly *Catenovulum* in one eye and *Corynebacterium* in the second eye (Figure 2A, Volunteer 2). A second volunteer had *Catenovulum* in one eye and a variety of bacteria in the other eye, including *Sphingomonas, Comamonas, Bradyrhizobium, Acinetobacter, Staphylococcus, Pseudomonas*, and *Mycoplasma* (Figure 2A, Volunteer 1). In an individual, we treated one eye as an independent microhabitat. All further analyses were performed at the level of a single eye, rather than a combination for each participant.

**Figure 3:**
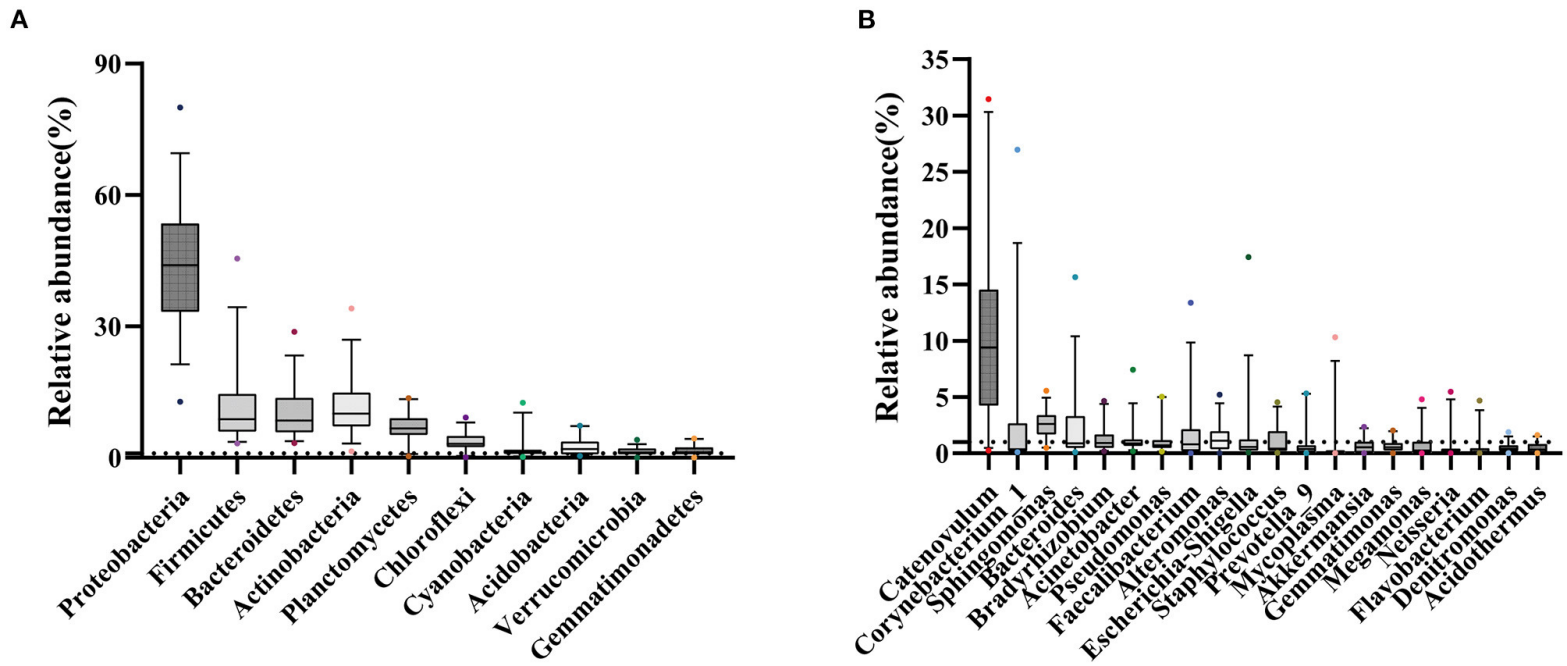
Sample distribution of relative abundance of bacteria at different taxonomic levels. **(A)** Top 10 at phylum levels. **(B)** Top 20 at genus levels.

At the phylum level, *Proteobacteria, Firmicutes, Actinobacteria*, and *Bacteroidetes* occupied the top four, which together accounted for 77.25% of all sequencing reads. They could be detected in all samples, and the relative abundance was more than 1% (Figure 3A; Figure S2). At the genus level, 11 bacterial genera had an average relative abundance of more than 1% in the population (Figure 3B). *Catenovulum* occupied the largest portion (0.2359–31.4431%), followed by *Corynebacterium* (0.1043–26.9518%), *Sphingomonas* (0.4794–5.5446%), *Bacteroides* (0.0879–15.6569%), *Faecalibacterium* (0.0268–13.3857%), *Alteromonas* (0.0179–5.2026%), *Escherichia-Shingella* (0.0812–17.4233%), *Bradyrhizobium* (0.1752–4.658%), *Acinetobacter* (0.1877–7.4161%), *Staphylococcus* (0.0398–4.523%), and *Pseudomonas* (0.1616–5.0249%). These bacteria were detected in the eyes of all participants, but the relative abundance values of different samples were significantly different. The proportion distribution of other bacteria was also different, but the range of relative abundance was smaller than that of the former two.

### Sodium Hyaluronate Eye Drops Altered Ocular Surface Bacterial Microbiota

**Figure 4:**
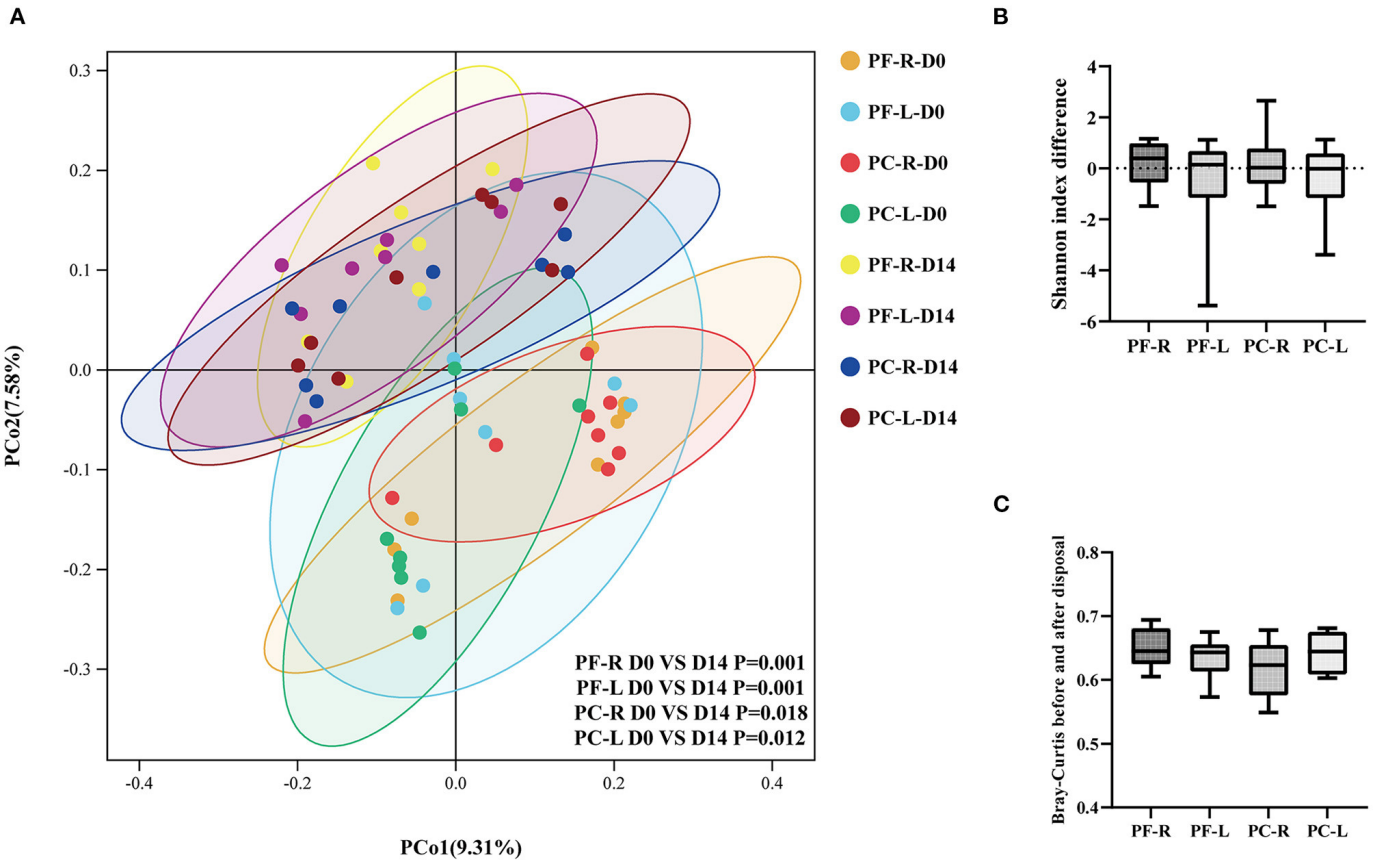
Alpha and beta diversity analysis within the individuals and between the preservative-free (PF) and preservative-containing (PC) groups. **(A)** The PCoA plot based on the unweighted UniFrac distance index. **(B)** The differences in Shannon index between the repeated samples in each group. **(C)** Bray-Curtis indices between the repeated samples in different groups.

Multivariate statistical analysis was used to observe the changes in the ocular surface bacterial microbiota before and after intervention with different doses of sodium hyaluronate eye drops in different groups. The PCoA plot showed the relationships of bacterial microbiota among individual samples. The higher the similarity between the two samples, the closer the distance of the straight line projected in the graph. The PCoA plot based on the unweighted UniFrac distance index showed that the bacterial community on the ocular surface changed significantly after the use of sodium hyaluronate eye drops (with or without BAC), whether within individuals or between individuals in different groups (Figure 4A, *P* < 0.05, PERMANOVA). We analyzed the alpha and beta diversities of each group before and after intervention. We calculated the differences in the Shannon index before and after intervention in each group and compared them between groups. Whether within individuals or between individuals in different groups using different disposal methods, the use of sodium hyaluronate eye drops (whether containing BAC or not) did not cause a significant change in alpha diversity (Figure 4B, *P* > 0.05, Kruskal–Wallis test). We calculated the distance index between the repeated samples. Whether within individuals or between individuals in different groups, the distance index changed significantly after the use of sodium hyaluronate eye drops (whether containing BAC or not). The average distance index of repeated samples in each group was more than 0.6. However, no significant difference was found among the groups (Figure 4C, *P* > 0.05, one-way analysis of variance). Combined with the results of Shannon index analysis, no significant change was noted in species richness before and after intervention. The reason for the large distance index between repeated samples was probably that the composition and proportion of bacterial communities in each sample had changed.

**Figure 5:**
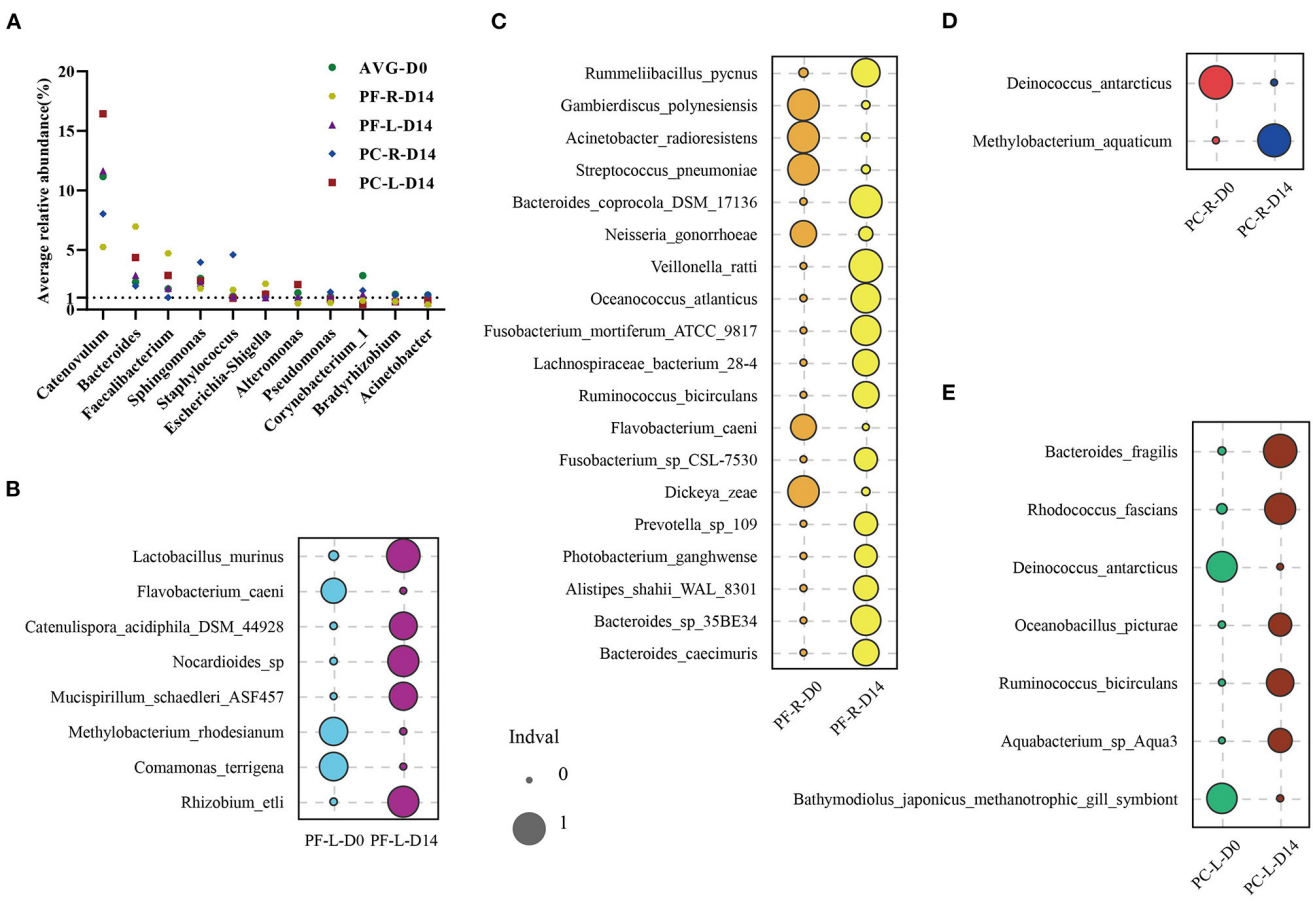
Indicator analysis of repeated samples from the preservative-free (PF) and preservative-containing (PC) groups. **(A)** Changes in the average abundance of 11 “core” bacterial genera in each group before and after intervention. Indicator analysis of **(B)** PF-L group, **(C)** PF-R group, **(D)** PC-R group, and **(E)** PC-L group.

To confirm our hypothesis, we first focused on 11 bacterial genera with an average relative abundance of more than 1% at baseline. These bacteria accounted for a relatively large proportion of the microbial community, and therefore the fluctuation in their relative abundance was bound to affect the composition of the whole bacterial community. We ranked the genera of bacteria in each group according to their relative abundance, as detailed in Table S4. These 11 genera of bacteria could be detected in all samples before and after intervention. The relative abundance of these bacteria was also ranked high among repetitive samples in different groups. Within and between individuals, the relative abundance of these bacteria fluctuated after the intervention of sodium hyaluronate eye drops (with or without BAC) (Figure 5A). Among these, four kinds of bacteria had an average relative abundance of more than 1% before and after the intervention; they were *Catenovulum, Bacteroides, Faecalibacterium*, and *Sphingomonas*. At the species level, we used the indicator analysis to find species with significant changes in relative abundance before and after intervention in different groups (Figures 5B–E; Table S4). The relative abundance of different species selected in each group was very low before and after intervention. The proportion of a few species was between 0.1 and 0.5%, and that of the rest was no more than 0.06%. A consistent and significant decrease in the relative abundance of *Flavobacterium caeni* in both eyes was found in the preservative-free group. In the preservative group, a consistent and significant decrease in intraocular relative abundance was observed with *Deinococcus antarcticus*.

## Discussion

Sodium hyaluronate eye drops are common artificial tears in China, accounting for about 60% of the dry eye market share, divided into single-dose and multi-dose packaging. Preservatives need to be added to conventional multi-dose artificial tears to prevent microbial growth. However, small bottles of artificial tears in single-dose packaging are discarded immediately after opening the bottle, and therefore preservatives are not required. The tear film and the ocular surface epithelial cells are the physical barriers between the eye and the environment. As a part of the ocular surface microenvironment, ocular surface symbiotic bacteria also play an important role in ocular surface health, and the change in dynamic balance may be related to ocular pathology. The present study hoped to provide guidance for rational drug use by evaluating the effects of sodium hyaluronate eye drops and their different dosage types (with or without preservatives) on ocular surface bacteria. The traditional bacterial culture methods have obvious limitations in identifying conjunctival microflora. In this study, 16S rDNA sequencing was used to compare the conjunctival sac bacterial microbiota between single-dose and multi-dose sodium hyaluronate eye drops before and after the intervention. Our study found that healthy people had a rich diversity of ocular surface bacterial microbiota, but the bacterial communities between the eyes were not completely consistent. We also found that sodium hyaluronate eye drops, whether containing BAC or not, caused changes in the ocular surface bacterial community after treatment. The changes in the ocular surface microenvironment caused by sodium hyaluronate eye drops with different composition seemed to inhibit some bacteria.

Although previous studies compared ocular surface microbiota between eyes, the conclusion was controversial: previous studies found no significant difference in alpha diversity between left and right eyes (7, 10, 15); however, the present study found significant differences in genera and the relative abundance. The bacterial communities on the ocular surface between eyes were not entirely similar. The study by Zilliox et al. (15) supported the conclusion of this study. They found that half of the healthy people and patients with ocular surface diseases had different microbiota between their eyes. However, still, some differences were noted between the present study results and previous reports. Wen et al. (7) found no significant difference in the composition of microbial communities between the left and right eyes of the same volunteer. In addition, no significant difference was observed in the relative abundance of “core” species. This inconsistency was probably due to the differences in samples and methods used. The age range of the sample population in this study was 24–84 years. The conjunctival imprinting cytology technique was used to collect microorganisms on the conjunctival surface of the lower hemisphere, and the DNA shotgun sequencing was used for follow-up analysis. The beta diversity was analyzed by PCA analysis. PCA analysis was a direct projection of the species abundance data in the sample. The distance in different groups of samples reflected the community differences between the groups. This analysis was based on a linear model. The so-called linear model assumed that species abundance changed linearly with the change in environmental variables. The scope of application of this model was limited. In real life, microbial abundance usually presents a unimodal model. Therefore, we thought that different raw data processing methods and statistical testing methods were also the reasons for the inconsistency between our conclusions and those of Wen et al. Differences existed in microbial communities among individuals, including many confounding factors. The limitation of our study and previous studies was in the lack of large–sample size data and data processing methods with gold standards. Larger–sample size research and recognized data research methods are needed to solve this controversial problem.

The core human microbiome is the set of genes present in a given habitat in all or the vast majority of humans (37). Dong et al. (3) identified the 10 most prevalent (defined as >1% of all detected genera) of the conjunctival microbiome, including *Pseudomonas, Bradyrhizobium, Propionibacterium, Corynebacterium*, and *Acinetobacter*. Zhou et al. (38) found 610 genera belonging to 22 phyla. Of the genera with a relative abundance > 1%, *Corynebacterium, Streptococcus, Propionibacterium, Staphylococcus, Bacillus*, and *Ralstonia* were present in 80% of the participants. Huang et al. (4) found that 10 bacterial genera that might represent the core genera accounted for more than 76% of the microbial community in the normal conjunctival sac. They were *Corynebacterium, Pseudomonas, Staphylococcus, Acinetobacter, Streptococcus Millisia, Anaerococcus, Finegoldia, Simonsiella*, and *Veillonella*. Ten genera were identified as common ocular bacteria in most participants (defined as >1% of all detected genera). Delbeke et al. (39) summarized the data on the ocular surface microbiome for a systematic review. In this study, the major phyla were found to be *Proteobacteria, Actinobacteria, Firmicutes*, and *Bacteroidetes*, with the first two being the most abundant. They defined the healthy adult core microbiome as genera present in minimum 5 out of the 11 published control cohorts with available raw data with a relative abundance of at least 1%. They found a core ocular surface microbiome comprising *Corynebacterium, Acinetobacter, Staphylococcus, Pseudomonas, Propionibacterium*, and *Streptococcus*. The present study also reported the core ocular surface bacterial microbiota in healthy adults. According to the definition in the previous literature, the core ocular surface microbiome, at the phylum level, includes *Proteobacteria, Firmicutes, Actinobacteria, Bacteroidetes, Planctomycetes, Chloroflexi, Cyanobacteria, Acidobacteria, Verrucomicrobia*, and *Gemmatimonadetes*. The relative abundance of the first four items accounts for the largest proportion, which was consistent with previous findings (4, 39). However, at the genus level, the results were slightly different from the previous ones. The discrepancy in this series of results was due to the differences in swab pressure, amplification region, primer selection, detection platform, and database (8). Current microbiome analysis techniques have been developed for samples with high bacterial biomass (such as feces or soil), but not for samples with low bacterial content (such as ocular surface). The lack of standardization of the research workflow limited the repeatability and reliability of the study. It is worth noting that Ozkan et al. (5) assessed the time stability of ocular surface microbiota by sampling 43 participants at 3 time points. No single OTU was shown to be present in all participants at all times or at any given point in time. The possibility of individual-specific (or “minimal”) core microbiomes was suggested. However, in our study, the 11 “core” genera were detected in all samples before and after intervention. Limited by the sample size, whether we have really defined the core ocular surface bacterial community of healthy people is debatable. Also, large differences in sample sizes existed in previous studies, and the conclusions thus obtained could not confirm the accurate definition of the core ocular surface microbiome. Future studies with larger sample sizes are needed to address this question.

Another finding of the present study was that the use of sodium hyaluronate eye drops, whether containing BAC or not, changed the ocular surface bacterial microbiota. Hyaluronic acid (HA) is a glycosaminoglycan polymerized by disaccharide units composed of glucuronic acid and N-acetyl glucosamine. It is the main component of the extracellular tissue and is abundant in synovial fluid, vitreous body, and aqueous humor of the joint cavity (40). Sodium hyaluronate is the second-generation product of HA. Using the first-generation common HA as the raw material, the low–molecular weight substances produced by mechanical, chemical, and biological enzyme-cutting methods are easier to absorb compared with the previous-generation products. The characteristic of sodium hyaluronate is that it can combine with ocular surface cells and form a regular, stable, and long-lasting water film on the ocular surface, which is not easy to wash off and promotes wound healing (41–44). The ocular surface microbiota settles on the mucosal epithelium of the ocular surface and the tear film covering it. The use of sodium hyaluronate eye drops resulted in a change in the microbial environment, which might be accompanied by an increase or decrease in nutrients. When two or more kinds of microorganisms have the same requirements for specific environmental factors, competition inevitably occurs. Certain bacteria that can adapt to the changes in the external environment stand out as the dominant species. Competition among species throughout the microecological environment creates this ebb and flow. It is also worth considering whether sodium hyaluronate itself is used as an energy source by some species to cause specific selection of microbial communities. Costagliola et al. (45) found that staphylococci and streptococci were obtained low–molecular weight sugars that could be used as nutrients by producing hyaluronidase in the presence of HA. Some bacteria such as *Pseudomonas aeruginosa* did not have the ability to produce hyaluronidase, thus limiting the benefit of sodium hyaluronate to them (46, 47). However, these results are not based on a complete microbiome, and therefore cannot explain whether this phenomenon occurs in the ocular surface microbiome.

Benzalkonium chloride (BAC) is used to avoid microbial contamination. It is also worth considering whether the environmental pressures imposed by BAC may cause some less tolerant species to fall behind in competition. However, how much it contributes to environmental change is unclear. The concentration of preservatives used in eye drops was very low, while the concentration of BAC in the present study was 0.003% (30 μg/mL). Tear dilution and blink scouring further weakened the effect of BAC. One study found that when 0.005% BAC (50 μg/mL) was injected into the eye for 1 min, the dilution multiple of BAC was 16 times, and the concentration of BAC after 5 min was negligible (48). BAC had neither the concentration nor the time (1 h) required to produce the antibacterial effect due to the rapid dilution of the tear film.

We analyzed differential species to explore whether single-dose and multi-dose sodium hyaluronate eye drops were selective for specific bacteria. According to the results of indicator analysis, no common and significant differences were found in 11 genera of “core” bacteria, either within individuals or between individuals in different groups. At the species level, most of the differential species selected in each group were those with an average relative abundance of <0.05%. In terms of relative abundance, we conjectured that even small fluctuations in bacteria with high values would have a significant impact on the values of the bacteria with low level in the bacterial community. However, sodium hyaluronate eye drops without BAC decreased the abundance of *F. caeni* between the eyes within the individual (*P* < 0.05). Meanwhile, the environmental pressure exerted by sodium hyaluronate eye drops containing BAC decreased the consistency of *D. antarcticus* between eyes. It seemed to suggest that these two specifications of sodium hyaluronate eye drops were selective for specific species (*P* < 0.05). However, it could not be ignored that the relative abundance of these two kinds of bacteria was not high; therefore, the reason for this phenomenon was likely to be consistent with our previous guess. Coupled with our sample size limit, we need more population samples for further research and analysis.

This study also had other limitations. First, gene sequencing could only detect the relative abundance of microorganisms and could not represent the true density of microorganisms in the environment. Hence, absolute quantitative indicators for the changes in the number of bacteria before and after the intervention were lacking, and the impact of differences in PCR amplification could not be ignored. Second, differences over the V3–V4 region were not enough to resolve everything to the species level. They were either identical or insufficiently different to identify a species within the genera. It limited the comprehensive analysis of the microbial community. Third, this study lacked the comparison of people of different ages. Hence, the impact of the same intervention on infants and even the elderly is worth in-depth exploration. Fourth, which environmental factors in the ocular surface microenvironment were changed by sodium hyaluronate eye drops was not clear. Fifth, what changes will occur once sodium hyaluronate eye drops are stopped or permanently used could not be predicted. We failed to indicate at what point in time the microbial community returned to the baseline level after stopping the use of sodium hyaluronate eye drops and whether the bacterial community really returned to it. Finally, the changes in ocular surface microbiota and the related mechanism in patients with different ocular surface diseases also needed to be further discussed.

In conclusion, 16S rDNA sequencing confirmed the rich diversity of the bacterial microbiota on the ocular surface of healthy people, but the bacterial communities between the eyes were not completely similar. Whether containing BAC or not, sodium hyaluronate eye drops could change the bacterial community on the ocular surface. These findings might help further understand the ocular surface bacteria and reasonably guide the use of drugs in the eye.

## Data Availability Statement

The raw sequence data were deposited into the Sequence Read Archive database (Accession Number: PRJNA720296).

## Ethics Statement

The studies involving human participants were reviewed and approved by the Ethics Committee of Xiamen Eye Center affiliated to Xiamen University. The participants provided their written informed consent to participate in this study.

## Author Contributions

CL and HW conceived and designed the experiments. YZ and XF collected samples and measured and analyzed data. XW and Y-AL analyzed the data. YZ wrote the manuscript. All authors read and approved the final manuscript.

## Funding

This study was supported in part by grants from the National Key R&D Program of China (Grant Numbers 2020YFA0908103 and 2018YFA0107301); the National Natural Science Foundation of China (Grant Numbers 82070931 and 81770891); and the Huaxia Translational Medicine Fund for Young Scholars (Grant Number 2017-A-001).

## Conflict of Interest

The authors declare that the research was conducted in the absence of any commercial or financial relationships that could be construed as a potential conflict of interest.

## Publisher's Note

All claims expressed in this article are solely those of the authors and do not necessarily represent those of their affiliated organizations, or those of the publisher, the editors and the reviewers. Any product that may be evaluated in this article, or claim that may be made by its manufacturer, is not guaranteed or endorsed by the publisher.
