# Supplementary material for: Effects of Sodium Hyaluronate Eye Drops With or Without Preservatives on Ocular Surface Bacterial Microbiota
Source: Front Med (Lausanne). 2022 Feb 14;9:793565. doi: 10.3389/fmed.2022.793565 (PMC8896347; doi:10.3389/fmed.2022.793565)
Supplement: Supplementary file 1 [file Data_Sheet_1.PDF]

Supplementary Material

Supplementary Figures

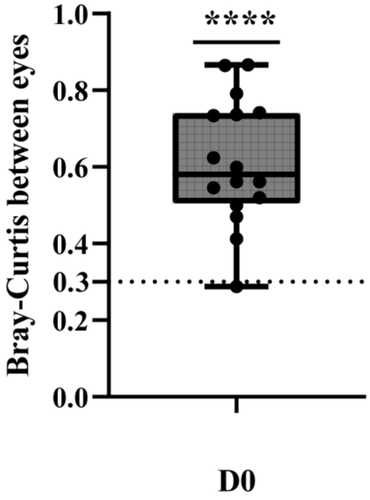

Supplementary Figure 1. Single-sample *t* test for distance index between eyes.

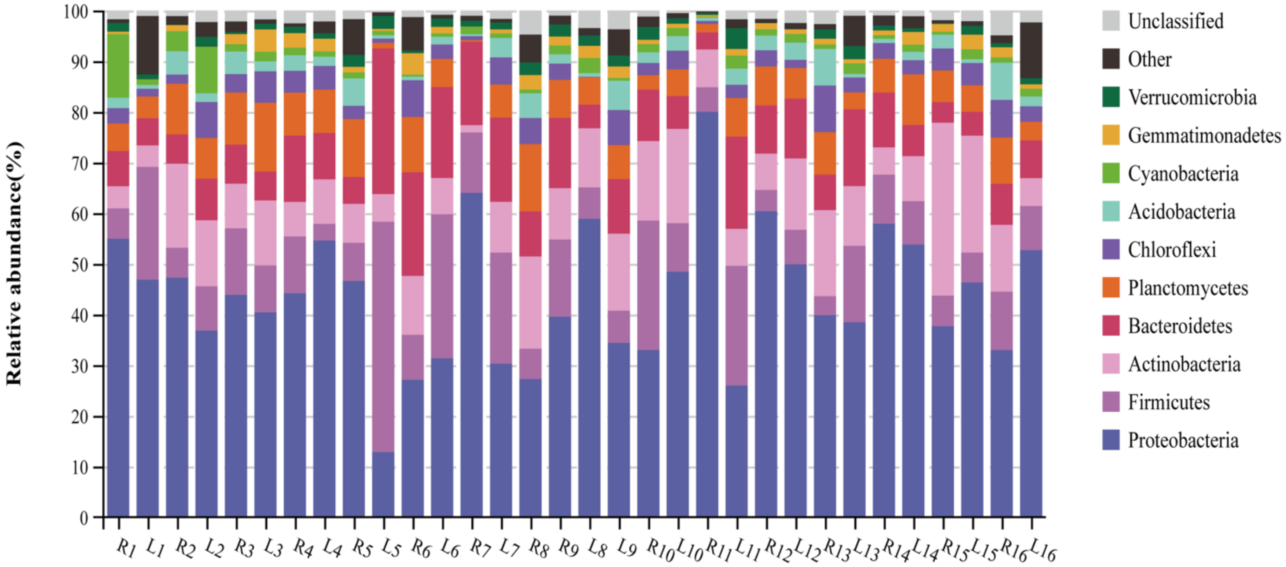

Supplementary Figure 2. Stacking map of abundance of bacteria at baseline. 16S rRNA gene sequences were classified into phylum levels.
