# Supplementary material for: Effects of Sodium Hyaluronate Eye Drops With or Without Preservatives on Ocular Surface Bacterial Microbiota
Source: Front Med (Lausanne). 2022 Feb 14;9:793565. doi: 10.3389/fmed.2022.793565 (PMC8896347; doi:10.3389/fmed.2022.793565)
Supplement: Supplementary file 4 [file Table_3.DOCX]

**Supplementary Table**

**Supplementary Table E3.**  **Data preprocessing statistics and quality control**

| **Sample Name** | **Raw PE** | **Clean tags** | **Effective tags** | **Effective ratio (%)** | **OTUs** |
| --- | --- | --- | --- | --- | --- |
| HYB1 | 109,963 | 104,280 | 95,037 | 86.43 | 1042 |
| HYB2 | 101,138 | 94,740 | 84,893 | 83.94 | 883 |
| HYA1 | 112,545 | 106,383 | 96,467 | 85.71 | 1415 |
| HYA2 | 114,954 | 108,424 | 95,450 | 83.03 | 1528 |
| HYB3 | 100,795 | 94,809 | 88,167 | 87.47 | 1022 |
| HYB4 | 100,918 | 94,561 | 90,173 | 89.35 | 993 |
| HYA3 | 109,624 | 102,861 | 90,787 | 82.82 | 1569 |
| HYA4 | 110,594 | 103,043 | 93,532 | 84.57 | 1515 |
| HYB5 | 106,175 | 99,943 | 91,282 | 85.97 | 983 |
| HYB6 | 114,265 | 107,126 | 99,658 | 87.22 | 1036 |
| HYA5 | 107,635 | 100,964 | 84,204 | 78.23 | 1400 |
| HYA6 | 109,313 | 102,760 | 94,813 | 86.74 | 1430 |
| HYB7 | 101,773 | 95,367 | 88,189 | 86.65 | 1025 |
| HYB8 | 113,482 | 105,735 | 95,034 | 83.74 | 1024 |
| HYA7 | 106,419 | 98,693 | 76,329 | 71.72 | 2127 |
| HYA8 | 112,166 | 104,936 | 94,140 | 83.93 | 1427 |
| HYB9 | 102,120 | 95,890 | 91,130 | 89.24 | 1094 |
| HYB10 | 105,489 | 96,747 | 81,168 | 76.94 | 1666 |
| HYA9 | 105,759 | 99,760 | 94,687 | 89.53 | 952 |
| HYA10 | 113,311 | 106,655 | 92,856 | 81.95 | 1938 |
| HYB11 | 105,411 | 98,581 | 89,544 | 84.95 | 1285 |
| HYB12 | 105,407 | 98,542 | 90,601 | 85.95 | 1241 |
| HYA11 | 101,002 | 94,991 | 87,006 | 86.14 | 1229 |
| HYA12 | 102,037 | 95,839 | 85,308 | 83.6 | 1368 |
| HYB13 | 113,222 | 101,774 | 67,266 | 59.41 | 674 |
| HYB14 | 110,414 | 103,003 | 94,073 | 85.2 | 1270 |
| HYA13 | 107,927 | 102,110 | 96,298 | 89.23 | 1003 |
| HYA14 | 104,599 | 98,202 | 91,249 | 87.24 | 1000 |
| HYB15 | 109,919 | 103,252 | 97,367 | 88.58 | 1057 |
| HYB16 | 104,488 | 98,553 | 90,998 | 87.09 | 968 |
| HYA15 | 102,392 | 96,204 | 87,979 | 85.92 | 1487 |
| HYA16 | 106,974 | 101,132 | 89,561 | 83.72 | 1206 |
| SHB1 | 113,670 | 106,497 | 96,160 | 84.6 | 1156 |
| SHB2 | 108,777 | 101,993 | 94,428 | 86.81 | 1116 |
| SHA1 | 105,873 | 99,529 | 83,647 | 79.01 | 1406 |
| SHA2 | 107,584 | 101,028 | 90,720 | 84.32 | 1505 |
| SHB3 | 104,085 | 97,843 | 85,072 | 81.73 | 1263 |
| SHB4 | 104,997 | 98,746 | 90,627 | 86.31 | 1076 |
| SHA3 | 113,569 | 106,435 | 97,541 | 85.89 | 1079 |
| SHA4 | 100,568 | 94,341 | 84,204 | 83.73 | 1437 |
| SHB5 | 109,608 | 103,246 | 96,813 | 88.33 | 954 |
| SHB6 | 103,019 | 96,557 | 88,614 | 86.02 | 1281 |
| SHA5 | 105,329 | 98,382 | 90,761 | 86.17 | 1114 |
| SHA6 | 114,388 | 107,953 | 97,040 | 84.83 | 1118 |
| SHB7 | 112,634 | 106,381 | 96,502 | 85.68 | 986 |
| SHB8 | 111,354 | 104,710 | 94,291 | 84.68 | 987 |
| SHA7 | 101,048 | 95,407 | 88,809 | 87.89 | 1022 |
| SHA8 | 109,915 | 103,199 | 94,798 | 86.25 | 1059 |
| SHB9 | 100,681 | 93,954 | 86,838 | 86.25 | 1223 |
| SHB10 | 110,190 | 102,683 | 93,540 | 84.89 | 1187 |
| SHA9 | 108,864 | 102,336 | 90,952 | 83.55 | 1526 |
| SHA10 | 112,279 | 105,135 | 94,540 | 84.2 | 1472 |
| SHB11 | 112,269 | 105,635 | 95,095 | 84.7 | 1236 |
| SHB12 | 100,145 | 93,883 | 85,067 | 84.94 | 1163 |
| SHA11 | 106,310 | 99,632 | 89,732 | 84.41 | 1066 |
| SHA12 | 103,571 | 97,008 | 87,085 | 84.08 | 1063 |
| SHB13 | 100,374 | 94,105 | 87,245 | 86.92 | 1064 |
| SHB14 | 108,326 | 101,186 | 94,584 | 87.31 | 1070 |
| SHA13 | 103,142 | 95,758 | 92,434 | 89.62 | 968 |
| SHA14 | 114,135 | 107,435 | 99,049 | 86.78 | 1231 |
| SHB15 | 107,061 | 99,695 | 93,259 | 87.11 | 1245 |
| SHB16 | 104,324 | 97,022 | 89,009 | 85.32 | 1105 |
| SHA15 | 114,822 | 108,933 | 101,717 | 88.59 | 1165 |
| SHA16 | 105,476 | 99,519 | 90,465 | 85.77 | 934 |
| Total | 6,870,617 | 6,442,026 | 5,825,884 |  | 77134 |
| Average | 107,353.40 | 100,656.66 | 91,029.44 | 84.83 | 1205.22 |
